# Unwinding the SARS-CoV-2 Ribosomal Frameshifting Pseudoknot with LNA and G-Clamp-Modified Phosphorothioate Oligonucleotides Inhibits Viral Replication

**DOI:** 10.3390/biom13111660

**Published:** 2023-11-17

**Authors:** Ekaterina Knizhnik, Stepan Chumakov, Julia Svetlova, Iulia Pavlova, Yuri Khodarovich, Vladimir Brylev, Vjacheslav Severov, Rugiya Alieva, Liubov Kozlovskaya, Dmitry Andreev, Andrey Aralov, Anna Varizhuk

**Affiliations:** 1 Lopukhin Federal Research and Clinical Center of Physical-Chemical Medicine of Federal Medical Biological Agency, 119435 Moscow, Russia; knizhnik.ek@phystech.edu (E.K.); j.i.svetlova@gmail.com (J.S.); pavlova.yuiv@gmail.com (I.P.); wws83@yandex.ru (V.S.); 2 Department of Biological and Medical Physics, Moscow Institute of Physics and Technology, 141701 Dolgoprudny, Russia; 3 Shemyakin-Ovchinnikov Institute of Bioorganic Chemistry, Russian Academy of Sciences, 117997 Moscow, Russia; hathkul@gmail.com (S.C.); khodarovich@mail.ru (Y.K.); v.brylev@yandex.ru (V.B.); cycloheximide@yandex.ru (D.A.); 4 Research and Educational Resource Center for Cellular Technologies of The Peoples’ Friendship University of Russia, 117198 Moscow, Russia; 5 Belozersky Institute of Physico-Chemical Biology, Lomonosov Moscow State University, 119992 Moscow, Russia; ruqiwa_eva@mail.ru; 6 Raman Spectroscopy Laboratory, Moscow Institute of Physics and Technology, 141701 Dolgoprudny, Russia; 7 Chumakov Scientific Center for Research and Development of Immune-and-Biological Products, Russian Academy of Sciences (Institute of Poliomyelitis), 108819 Moscow, Russia; lubov_i_k@mail.ru

**Keywords:** SARS-CoV-2, ribosomal frameshifting, oligonucleotides, modification, antivirals, locked nucleic acids, gapmers, phenoxazine

## Abstract

Ribosomal frameshifting (RFS) at the slippery site of SARS-CoV-2 RNA is essential for the biosynthesis of the viral replication machinery. It requires the formation of a pseudoknot (PK) structure near the slippery site and can be inhibited by PK-disrupting oligonucleotide-based antivirals. We obtained and compared three types of such antiviral candidates, namely locked nucleic acids (LNA), LNA–DNA gapmers, and G-clamp-containing phosphorothioates (CPSs) complementary to PK stems. Using optical and electrophoretic methods, we showed that stem 2-targeting oligonucleotide analogs induced PK unfolding at nanomolar concentrations, and this effect was particularly pronounced in the case of LNA. For the leading PK-unfolding LNA and CPS oligonucleotide analogs, we also demonstrated dose-dependent RSF inhibition in dual luciferase assays (DLAs). Finally, we showed that the leading oligonucleotide analogs reduced SARS-CoV-2 replication at subtoxic concentrations in the nanomolar range in two human cell lines. Our findings highlight the promise of PK targeting, illustrate the advantages and limitations of various types of DNA modifications and may promote the future development of oligonucleotide-based antivirals.

## 1. Introduction

**Figure 1:**
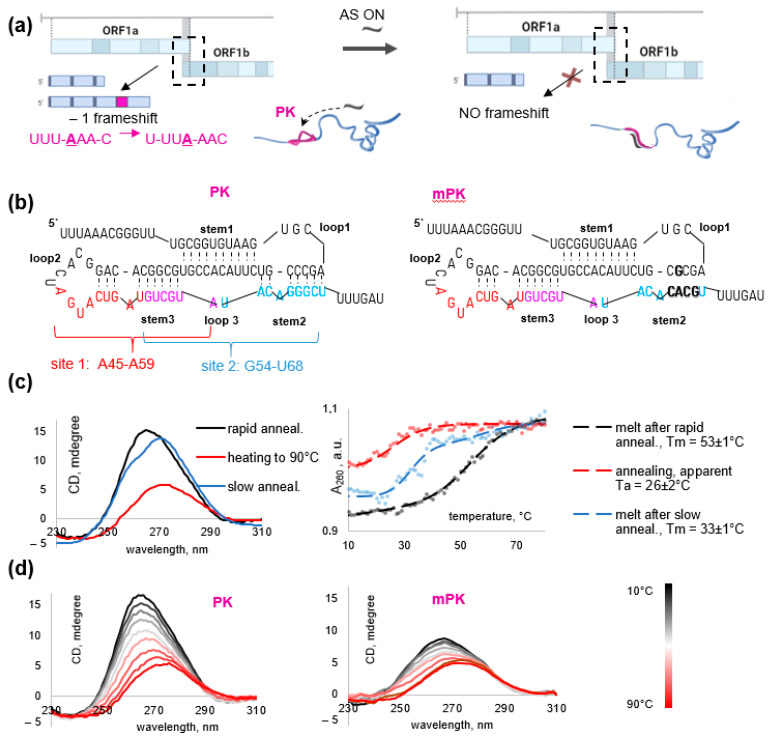
SARS-CoV-2 pseudoknot: function, structure, and potential target sites. (**a**) Schematic representation of pseudoknot (PK)-dependent RFS upon SARS-CoV-2 ORFab translation (left panel) and its expected attenuation in the presence of PK-disrupting AS ONs (right panel). (**b**) Schematic representation of the secondary structure of PK (left panel) and its mutant mPK (right panel). Potential target sites are marked. Mutated nucleotides in mPK are in bold font. (**c**) The impact of the annealing rate on PK folding evidenced by CD spectra (left) and UV-melting curves (right). Conditions: 1 µM RNA solutions in 10 mM sodium phosphate buffer supplemented with 100 mM KCl. (**d**) Comparison of PK and mPK CD-melting. Conditions in (**c**,**d**): 1 µM RNA solutions in 10 mM sodium phosphate buffer supplemented with 100 mM KCl).

Intense efforts have been made to combat coronavirus disease 19 caused by severe acute respiratory syndrome-related coronavirus 2 (SARS-CoV-2). The need for effective antivirals has stimulated the studies of the viral transcription–replication machinery [1], with a particular focus on its inhibitors or the inhibitors of its biosynthesis. The transcription–replication complex of SARS-CoV-2 comprises several non-structural proteins (NSPs) produced by the proteolytic cleavage of the precursor polyproteins pp1a (NSP1-11) and pp1ab (NSP1-16). These precursor polyproteins are encoded by the overlapping reading frames ORF1a and ORF1b [2]. Translation of the latter frame requires a programmed -1 ribosomal frameshift (RFS) at the UUUAAAC slippery site of the SARS-CoV-2 RNA [3] (Figure 1a). RFS is facilitated by the downstream pseudoknot structure (PK), which has to be unfolded before entering the ribosomal mRNA channel. This effect is partly counterbalanced by the upstream attenuator loop, leading to an overall frameshifting efficiency between 25 and 70%. A combination of the slippery site, the 5′ attenuator loop, and the three-stem 3′ PK is typical of all coronaviruses [4].

Studies of the SARS-CoV-2 PK structure by Cryo-EM [5], crystallography [6,7], and computational approaches [8,9] have revealed several possible conformers, and their dynamics has been clarified to some extent using molecular tweezers [10,11]. Major conformers include the ring-knot ones in which the 5′-end is threaded through the three helices and those without threading. RSF supposedly requires the coexistence of these two ensembles of conformers. Thus, shifting the equilibrium to either stabilized threaded conformers or unthreaded ones may affect the NSP1-11:NSP1-16 ratio and eventually disrupt the viral life cycle [4,12]. The respective drug design strategies include targeting the PK ring-knot pocket with small molecules [13,14,15] or stem-loops with CRISPR-Cas genome editing tools [12] or antisense (AS) oligonucleotides (ONs) [5,16] (Figure 1a). In the latter approach, the rationale for selecting target sites (particular PK stems) is a matter of debate.

Stem 1 appears to be the most stable and refolds quickly after PK disruption [10], while stems 2 and 3 are relatively mobile and fold after stem 1. On the one hand, such a hierarchy implies a particular importance of stem 1, which encourages targeting it with AS ONs. On the other hand, it suggests an enhanced exposure of stem 2/3-forming PK fragments in the single-stranded form, highlighting them as easily accessible targets. The first generation of coronavirus RFS inhibitors included peptide nucleic acids (PNAs) designed to invade stems 2 and 3 of SARS-CoV-1 PK [17], which is almost identic to that of SARS-CoV-2. Those PNAs suppressed viral replication at micromolar concentrations. Recently, SARS-CoV-2 PK-targeting gapmers containing LNA residues and phosphorothioate internucleotide linkages (PSs) were reported [5]. Those designed to disrupt stem 1 were active at high nanomolar concentrations in both frameshifting assays and pseudoviral replication assays, while several of the tested stem 2/3-disruptors turned out to be inefficient.

To clarify whether AS ONs performance in frameshifting assays is reflective of their ability to disrupt PK folding, we readdressed stems 2 and 3 of SARS-CoV-2 PK, taking into account their predicted propensity for rearrangements. Three types of stem 2/3-targeting AS ONs, namely LNA gapmers (LDL), fully modified LNA, and G-clamp-modified ONs with PS linkages (CPS), were obtained. We assessed their effects on PK structure using electrophoretic mobility shift assays (EMSAs) and circular dichroism (CD) spectroscopy. Next, we verified inhibitory activities of the AS ONs using luciferase reporter system-based frameshifting assays. Finally, we compared EMSA and DLA data and tested selected leaders against SARS-CoV-2 in two cell lines.

## 2. Materials and Methods

### 2.1. Pseudoknot RNA and Antisense Oligonucleotide Analogs

To select optimal target sites within SARS-CoV-2 PK, the homology between PK fragments and host (human) RNA fragments was analyzed using the NCBI blast suite and RefSeq RNA database. The thermal stability of presumed PK-AS ON duplexes (200 nM) at a physiological solution ionic strength (150 mM) was predicted using the R. Penchovsky TM calculator [18].

T7 promoter-containing dsDNA templates for wild-type (PK) and mutant (mPK) SARS-CoV-2 RNA were obtained from short synthetic ONs pk1–3 in 2 PCR steps. Pk1–3 were purchased from Litekh (Moscow, Russia). 

pk1: TAATACGACTCACTATAGGGAGAGTTTTTAAACGGGTTTGCGGTGTAAGTGCAGCN (PK, N = C; mPK, N = G);

pk2: CGTCTTACACCGTGCGGCACAGGCACTAGTACTGATGTCGTATACANNNNTTTTGAT (PK, NNNN = GGGC; mPK, NNNN = CACG);

pk3: GCACGGTGTAAGACGGGCTGCACTTACACC;

forward primer: TAATACGACTCACTATAGGGAG;

reverse primer: ATCAAAAGCCCTGTATACGAC.

Prior to step 1, ON pk2 was phosphorylated with T4 PNK (New England Biolabs, Ipswich, MA, USA). Step 1 PCR with pk1, phosphorylated p2, and pk3 was performed using PfuSE polymerase (Evrogen, Moscow, Russia) and gave a template for step 2 PCR, which was performed using Taq polymerase (Evrogen, Moscow, Russia). 

Sense strand of the resulting PK dsDNA: 

TAATACGACTCACTATAGGGAGAGTTTTTAAACGGGTTTGCGGTGTAAGTGCAGCCCGTCTTACACCGTGCGGCACAGGCACTAGTACTGATGTCGTATACAGGGCTTTTGAT (the T7 promoter is underlined);

Sense strand of the resulting mPK dsDNA:

TAATACGACTCACTATAGGGAGAGTTTTTAAACGGGTTTGCGGTGTAAGTGCAGCGCGTCTTACACCGTGCGGCACAGGCACTAGTACTGATGTCGTATACACACGTTTTGAT (the T7 promoter is underlined).

PK and mPK RNA were obtained from dsDNA templates as described previously [19]. Briefly, 2–3 µg DNA was used for the in vitro transcription with a HiScribe™ T7 High Yield RNA Synthesis Kit following the manufacturer’s protocol. The reaction mixture was treated with RNase-free DNAse I, and PK RNA was precipitated from cold ethanol. AS ONs (purity ≥ 95%, HPLC) were obtained from Litekh (Moscow, Russia).

### 2.2. Optical Methods and Electrophoretic Mobility Shift Assays (EMSA)

For secondary structure verification by optical methods, 1 µM RNA solutions in 10 mM sodium phosphate buffer supplemented with 100 mM KCl were annealed rapidly (heated to 90 °C and then snap-cooled on ice) or slowly (heated to 90 °C and then cooled gradually to room temperature) prior to all experiments. Circular dichroism (CD) spectra were registered at room temperature unless otherwise specified using a Chirascan spectrophotometer (Applied Photophysics, Leatherhead, UK) and a 1 cm optical path quartz cuvette. Melting/annealing experiments were also performed using a Chirascan spectrophotometer with a heating/cooling rate of 1 °C/min. 

For CD titration assays, AS ONs were added to the 1 µM PK solution to a final concentration of 0–1.5 µM. The CD spectra of pure AS ONs were used to evaluate their molar ellipticity at 260 and 272 nm and calculate the theoretical CD amplitudes of the non-bound AS-PK mixtures. A comparison of the theoretical and experimental CD spectra was summarized as a CD amplitude ratio vs. concentration plot.

For EMSA, 0.5 µM RNA solutions were mixed with AS ONs (final concentrations: 0–5 µM), incubated for 15 min and loaded onto 10% nondenaturing polyacrylamide gel. The gel was run at room temperature using a standard 1 × TBE buffer and stained with SYBR Green II. Relative intensities of folded and unfolded PK bands were measured using ImageJ 1.49 software and used to evaluate Kd values by fitting the experimental dependence of the unfolded PK fraction on the AS ON concentration to Equation (1):

Fraction = [AS ON]/([AS ON] + Kd) (1)

At least 2 EMSA repeats were performed for each AS ON.

### 2.3. Cell Cultures, Viability Assays, Flow Cytometry and Fluorescence Microscopy

The angiotensin-converting enzyme 2 (ACE2)-expressing HEK-293T-ACE2 cell line was established previously [20] from HEK-293T cells (ATCC CRL-3216, human epithelial-like cells), and the GBM6138 cells (ACE2-expressing glioblastoma multiforme cell line [21]) were a kind gift from Peter Chumakov. Cells were cultured in DMEM-F12 medium supplemented with 10% fetal bovine serum (FBS), 2 mM L-alanyl-L-glutamine, 100 U/mL penicillin, and 100 µg/mL streptomycin. To assess cell viability, the cells were plated in 96-well plates and transfected with AS ONs using Attractene transfection reagent (Qiagen, Germantown, MD, USA) following the manufacturer’s protocol. The old medium was replaced with 50 µL of CellTiter GLO 2.0 reagent (Promega, Madison, WI, USA) per well 24 h, 48 h, or 4 days after transfection. The plates were then incubated in the dark for 5 min, after which luminescence levels were quantified using a Triad microplate luminometer (Dynex, Pewaukee, WI, USA).

For intracellular localization studies, AS ON analogs labeled at the 5′-terminus with 6-carboxyfluorescein (FAM) were used; the cells were fixed 24 h after transfection, and the nuclei were stained with DAPI (Thermo, Waltham, MA, USA). Fluorescence microscopy imaging was performed using a Nikon Eclipse Ti2 microscope (Nikon, Tokyo, Japan).

### 2.4. Dual Luciferase Reporter System and Frameshifting Assays

Three previously described pSGDlucV3.0 vector-based dual luciferase expression constructs [3] were a kind gift from Gary Loughran (University College Cork). Construct WT contained a wild-type SARS-CoV-2 fragment with the attenuator loop, the slippery site, and PK between Renilla and Firefly luciferase genes. Construct SSmut (negative control) contained an RFS-preventing mutation in the slippery site. In construct IFC (in-frame control), the slippery site was absent, and the frame was shifted to ensure the expression of both luciferases. 

HEK-293T cells were plated in 96-well plates at a seeding density of 3 × 10^4^ viable cells per well. The next day, the cells were transfected with a WT/SSmut/IFC plasmid or its mixture with AS Ons (plasmid:AS ON mass ratio—up to 1:1). The transfection complexes for one well transfection were prepared as follows: Attractene (0.75 μL) was added to 200 ng of DNA in 50 μL Opti-Mem (Gibco) and incubated for 15 min. After adding transfection complexes to the wells, the cells were grown at 37 °C in 5% CO_2_ for 24 h. Then, the cells were lysed in 15 μL of 1× passive lysis buffer (Promega, Madison, WI, USA).

Renilla and Firefly luciferase substrates (50 µL) were added to each lysate sample. The substrates were purchased from Promega (USA). Light emission at 480 nm (Renilla) and 560 nm (Firefly) was measured using a M200 Tecan microplate reader (Tecan Group Ltd., Mannedorf, Switzerland). RFS efficiencies in WT/SS mut constructs were calculated as a ratio of Firefly (emission at 560 nm) and Renilla (emission at 480 nm) luciferase activities divided by the respective values in the IFC construct. All RFS values in the presence of AS ONs are shown as a percentage of RFS in the absence of AS ONs. The IC_50_ values were obtained by fitting the dose-dependent data to Equation (1).

### 2.5. Viral Replication Assays

HEK-293T-ACE2 and GBM6138 cells were cultured in 96-well plates at densities of 2 × 10^4^ and 5 × 10^4^ cells per well, respectively. Cells were transfected with AS ONs using Attractene transfection reagent in accordance with the manufacturer’s guidelines. Specifically, 3.75 µL of Attractene was used per µg of AS ON, mixed in 50 µL of serum-free medium, and applied to each column in two-fold dilutions. The cells were then incubated at 37 °C with 5% CO_2_ for 3 h. Subsequently, 50 µL of SARS-CoV-2 virus suspension (strain PIK35 (GISAID ID EPI_ISL_428852) obtained from FSASI “Chumakov FSC R&D IBP RAS” (Institute of Poliomyelitis, Moscow, Russia) was added to each well, starting at a concentration of 0.00025× of the original stock (1 × 10^6^ TCID_50_), with two-fold dilutions across rows, in triplicate. After a 2 h incubation, 100 µL of medium containing 2% FBS was added to each well. To directly measure the impact of AS ONs on viral titers, cells were incubated for an additional 5 days at 37 °C with 5% CO_2_, which was followed by a visual assessment of the cytopathic effect (CPE). To evaluate the rate of viral replication, media aliquots were collected on the third day of incubation and used to infect fresh cells under identical conditions. The TCID_50_ values were calculated using the Kärber method, as outlined in the referenced study [22]. EC_50_ values were calculated using Prism 9 (GraphPad, Boston, MA, USA). The impact of AS ONs on the viral replication rate in GBM6138 cells was additionally verified by RT-qPCR. RNA was extracted from the media aliquots obtained from the transfected cells on day 3 after infection (i.e., the same aliquots that were used in CPE assays) and subjected to RT-qPCR using the Polyvir SARS-CoV-2 kit (Litekh, Moscow, Russia). Prior to that, the kit was calibrated using a series of two-fold dilutions of PIK35 SARS-CoV-2 suspension (1 × 10^6^ TCID_50_ stock).

## 3. Results and Discussion

### 3.1. PK Folding Control and Selection of the Target Sequences

To select AS ONs that disrupt SARS-CoV-2 PK stems and unfold the overall PK structure, we first obtained a 96-nt PK-forming fragment of viral RNA (Figure 1b) and verified its folding by CD spectroscopy (Figure 1c). The CD spectrum of the rapidly annealed PK contained an A-form signature, namely a strong positive band at 260–265 nm, suggesting the presence of dsRNA fragments [23]. After slow annealing, the major band was shifted and its amplitude was reduced slightly, which is indicative of the increased ssRNA contribution with a major positive band at approximately 272 nm and a minor negative band at 240 nm [24].

In line with the CD data, UV melting of the rapidly annealed sample confirmed the presence of a folded structure that sustained the physiological temperature (Tm > 37 °C, Figure 1c), while the slowly annealed sample showed decreased hypochromism at 260 nm and gave an obscure melting curve with an apparent Tm value close to 37 °C. Therefore, we used rapid annealing in all subsequent experiments with AS ONs. Substantial hysteresis (>20 °C) was attributed to the conformational polymorphism of PK and its sequential folding [10].

In addition to the native PK, we obtained its analog—mPK with five mutations that disrupt stem 2 (Figure 1b) to verify the difference in electrophoretic mobility between folded and partially or totally unfolded RNA. While most mutations found in native SARS-CoV-2 variants allow for alternative knot-like structures (e.g., due to pairing between the former stem 2 and loop 3 or the 5′-overhang) [25,26], the mutations selected for mPK have been predicted to exclude alternative knots. Unlike PK, mPK was at least partially unfolded even in the absence of AS ONs according to CD spectroscopy data (Figure 1d).

Next, we compared PK stem-loop fragments as possible target sites for AS ONs in terms of homology to human mRNAs, which determines the potential side effects of AS ONs. Each SARS-CoV-2 PK stem shares similarity with at least one human transcript. Stems 1 and 3 are homologous to the fragments of mRNA encoding an anti-inflammatory protein TSC22D3 and an arginine methyltransferase CARM1, respectively. Top-scoring alignments of stem 2 include OSTF1 (osteoclast-stimulating factor 1), BAIAP3 (protein-binding partner of BAI1, which encodes brain-specific angiogenesis inhibitor), and USP18 (ubiquitin-specific peptidase 18). We concluded that minor interference with human transcripts cannot be excluded in any case and focused on 15 nt targets whose similarity with human mRNA fragments does not exceed 13 nt.

Finally, we compared PK fragments in terms of accessibility for AS ONs and the thermal stability of the respective duplexes. Due to the presence of bulges, stems 2 and 3 are easier to disrupt than stem 1, so we focused on the PK fragment that spans stem 2, loop 3 and stem 3. Within this region, we selected sites 1 and 2 (Figure 1b) with medium G/C content (40% and 53%, respectively). Their hypothetical duplexes with native AS ONs show comparable predicted melting temperatures well above the physiological value (45 °C and 49 °C, respectively). Both sites overlap with the junction between stem 3 and stem 1. Disruption of this junction is supposed to prevent the formation of any threaded PK conformers. Thus, we selected PK sites 1 (A45-A59) and 2 (G54-U68) as targets for AS ONs. Previously, the G54-U68 site of SARS-CoV-1 PK, almost similar to that in SARS-CoV-2, has been successfully targeted with AS PNAs in vitro [27].

### 3.2. Design of AS ONs and Comparison of Their PK Unfolding Potential In Vitro

**Figure 2:**
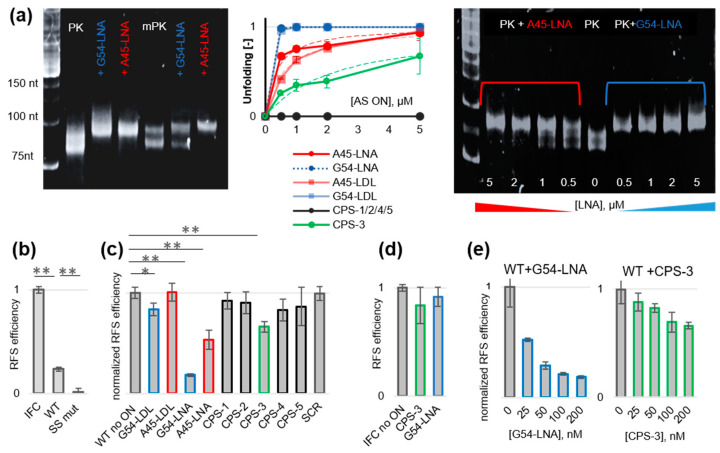
Pseudoknot unwinding by AS ONs: electrophoretic mobility (**a**) and dual luciferase assays (**b**–**e**). (**a**) Electrophoretic mobility assays. Left panel: PAGE of PK, mPK, and their complexes with site 1-targeting (red) and site 2-targeting (blue) LNA. PK/mPK concentration: 0.5 µM; LNA concentration: 2 µM. Middle panel: PAGE-based summary of PK unfolding by LNA, LDL and CPS AS ONs. Right panel: PAGE of PK (0.5 µM) in the presence of varying LNA concentrations. (**b**) Dual luciferase assays: frameshifting efficiency in constructs with a wild-type SARS-CoV-2 slippery site and PK (WT), in-frame positive control (IFC), and a negative control with mutated slippery site (SS mut) between the luciferase genes. (**c**) Effects of 200 nM AS or scramble (SCR) ONs on WT frameshifting efficiency. (**d**) Effects of the two AS ON leaders (200 nM) on IFC frameshifting efficiency. (**e**) Dose-dependent effects of the two leaders on WT frameshifting efficiency. All data in (**a**–**c**) are mean values of three biological repeats with three technical repeats each. Error bars are SD values. * *p* < 0.05 by Student’s two-tailed *t* test; ** *p* < 0.01. In (**d**), the data are presented as the mean of three technical repeats (SD did not exceed 10%).

We designed three sets of AS ONs targeting PK sites 1 and 2 (Table 1): LNA oligomers G54-LNA and A45-LNA; gapmers G54-LDL and A45-LDL, and phosphorothioate oligomers with phenoxazine-based G-clamp nucleoside insertions (CPS1–5). LNA backbone modification prevents nuclease digestion and enhances the thermal stability of ON-RNA duplexes [28]. Total LNA modification also hampers the recognition of the ON-RNA duplexes by RNase H, while the presence of a native central fragment (≥7 nt) in LDL gapmers restores the recruitment of RNase H [28]. Phosphorothioate internucleotide modification improves nuclease resistance, does not prevent the recruitment of RNase H, and has a minor effect on duplex stability [28,29,30]. The substitution of a native 2′-deoxycytidine residue for a four H-bond-forming G-clamp nucleoside improves mismatch discrimination and increases nuclease resistance [31] and duplex stability in a sequence-dependent manner without hampering the activation of RNase H [32]. Thus, we expected LNA, LDL, and CPS ONs to be protected from nuclease hydrolysis and unwind PK due to efficient binding with the target RNA. LDL and CPS ONs may both unwind PK and activate RNase H [28,33]. The PK unfolding potential of all AS ONs was tested using EMSA (Figure 2a, Figure S3).

First, we compared PK and mPK to verify the difference between mostly folded (PK) and mostly unfolded (mPK) variants (Figure 2a, left panel). Both gave two close bands in EMSA, and mPK was slightly less mobile than PK. In the case of PK, we attribute the two bands to threaded and unthreaded or folded and partially unfolded conformers. In the case of mPK, the two bands may correspond to partially and totally unfolded conformers. SYBR Green II stained PK more efficiently than mPK, which agrees with the larger number of dsRNA elements in PK. The electrophoretic mobility of PK was reduced in the presence of the excess of site 1-targeting (A45-LNA) or site 2-targeting (G54-LNA) AS ONs. Four equivalents of AS ON resulted in a complete loss of the lower band, indicating an utter transition to a less compact structure. This result is consistent with our assumption that both types of ONs unwind PK. In contrast, the lower band of mPK disappeared only in the presence of the site 1-targeting AS ON A45-LNA, while the effect of site 2-targeting G54-LNA was minor because of the mutated site 2 in mPK. To summarize, we showed that the disappearance of the lower band in EMSA indicates PK unfolding.

Next, we performed the same assays with all sets of AS ONs and analyzed concentration dependence for the preliminarily evaluation of EC50 values (Figure 2, middle panel). AS ONs from the CPS set failed to unwind PK except for the site 2-targeting with two closely positioned G-clamp residues—CPS-3 (the EC50 value was in the low micromolar range). In all AS ON sets, site 2-targeting ONs were generally superior to site 1-targeting ones. This trend may result from the difference in the thermal stability of respective RNA-AS ON duplexes or the enhanced accessibility of site 2, considering that stem 2 forms after stem 3 upon PK folding [10]. LNA and LDL gave comparable results (the EC50 values were in the submicromolar range), but A54-LNA was slightly superior to A54-LDL. The representative electropherogram illustrating LNA effects is shown in the right panel of Figure 2a and the electropherograms with other AS ONs are shown in Figure S1. LNA effects were additionally verified by CD titration assays, and the results were in line with EMSA (Figure S2). Although unfolded PK fractions could not be estimated directly because of the excess LNA contribution to the ellipticity, the spectra of the mixtures were clearly distinct from those predicted for unbound PK and LNA, supporting PK rearrangement at submicromolar LNA concentrations.

### 3.3. Inhibitory Activity of the AS ONs in Frameshifting and Viral Replication Assays

The ability of AS ONs to inhibit RFS was evaluated using dual luciferase assays (DLAs). For that, we co-transfected HEK-293T cells with AS ONs and the WT dual luciferase plasmid (attenuator + wild-type slippery site + PK of SARS-CoV-2 between the Renilla and Firefly luciferase genes) or the IFC (in-frame control) plasmid and calculated RFS efficiency based on the Firefly/Renilla luciferase activities in cellular lysates. In the absence of the AS ONs, the RFS efficiency in WT samples was equal to 25 ± 3%, which agrees well with previous report [3], while the negative control plasmid SS Mut with a mutated slippery site showed negligible RFS (Figure 2b). For initial screening, AS ONs were used at a concentration of 200 nM. The site 1-targeting AS ON A45-LNA and the site 2-targeting AS ONs G54-LDL, CPS-3, and G54-LNA caused a statistically significant reduction in RFS efficiency (Figure 2c). The scramble sequence (SCR) [5] had no significant effects. The LDL AS ONs were inferior to the LNA ones, which agrees qualitatively with EMSA data. In line with EMSA data, site 2-targeting AS ONs were superior to site 1-targeting ones with analogous modifications. In particular, the effect of G54-LNA (RSF reduction by 82 ± 2%) was 1.5 times stronger than that of A45-LNA (RSF reduction by 50 ± 10%). Therefore, we focused on site 2-targeting AS ONs in subsequent studies.

Relative effects of LNA and CPS AS ONs in DLA were slightly different from those observed in EMSA. In particular, the advantage of LNA over LDL gapmers was more pronounced, and CPS-3 outperformed G54-LDL (Figure 2c). This result may be explained by the enhanced nuclease resistance of AS ONs with total backbone modifications (LNA and CPS) as compared to the LDL gapmers. For subsequent experiments, we selected two totally modified AS ONs inducing statistically significant (*p* < 0.01) WT RSF changes at a concentration of 200 nM (Figure 2c): G54-LNA and CPS-3. Importantly, G54-LNA had no significant effect on IFC RSF, while CPS-3 induced a minor decrease in ICF RSF (Figure 2d). These data support PK unwinding as a primary mechanism of action of G54-LNA, while CPS-3 might additionally activate RNase H and induce its degradation in addition to PK unfolding. The effects of both AS ONs showed concentration dependence (Figure 2e), but only G54-LNA was active in the medium nanomolar concentration range (EC50 ≈ 26 ± 9 nM).

The leading AS ONs G54-LNA and CPS-3 were further tested against SARS-CoV-2 using cytopathic effect (CPE) assays. For that, ACE2-expressing HEK-293T (HEK-293T-ACE2) and GBM6138 glioblastoma cells were used. The former was selected as one of the most common non-tumor model cell lines in SARS-CoV-2 assays [34]. The latter was added to account for the presumed susceptibility of cancer cells to SARS-CoV-2 infection [35]. GBM6138 cells are particularly susceptible to SARS-CoV-2 due to the deficient production of interferon type 1 and overexpression of ACE2 [36]. In the future, additional tests with epithelial and/or respiratory tissue-derived cells may be required for the comprehensive characterization of the AS ONs. However, such cells tend to show heterogeneity in ACE2 or cell surface protease levels [37], whereas the model cell lines with ectopic ACE2 expression are reproducibly infection permissive. 

The cells were transfected with AS or control (SCR) ONs at final concentrations up to 200 nM; then, they were treated with the SARS-CoV-2 suspension and incubated for 5 days prior to CPE analysis. In parallel to that, the viability of non-treated cells was evaluated 4 days after transfection with the ONs. Intracellular localization of the ONs was examined by fluorescence microscopy imaging 24 h after transfection using 10% admixtures of 5′-FAM-labeled ON analogs. G54-LNA and SCR showed analogous intracellular distribution: both tended to localize in the cytoplasm with occasional entry into the nucleus in HEK-293T-ACE2 cells. CPS-3 was observed only in the cytoplasm (Figure S3a). In glioblastoma cells, all AS ONs accumulated in the cytoplasm but showed uneven distribution, suggesting possible off-target interactions with host proteins or RNA (Figure S3b).

**Figure 3:**
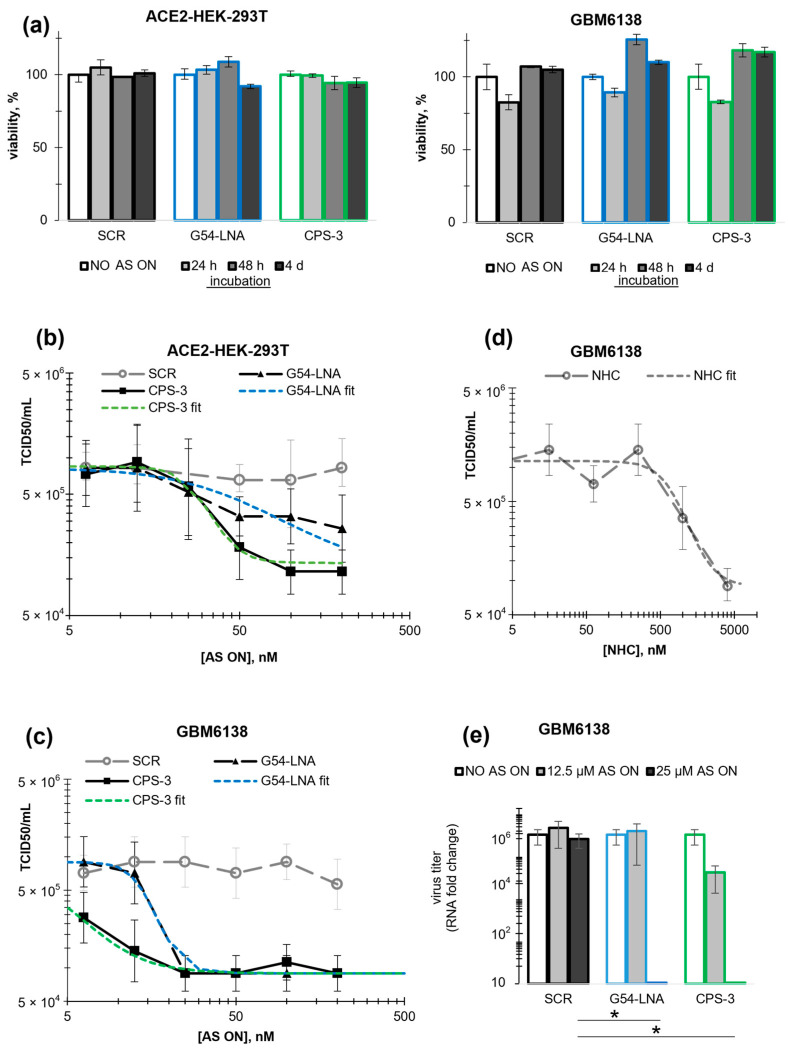
Toxicity and antiviral activity of AS ONs. (**a**) Toxicity of AS ONs toward ACE2-expressing HEK-293T-ACE2 cells (left) and GBM6138 glioblastoma cells (right). The percentage of metabolically active cells (viability) was evaluated based on the ATP present 24 h, 48 h, or 4 days after transfection with AS ONs or blank solutions. (**b**) Antiviral activity of AS ONs in HEK-293T-ACE2 cells. Direct virus titer. (**c**) Antiviral activity of AS ONs in GBM6138 cells. Secondary virus titer. (**d**) Activity of the control small molecule, NHC, in GBM6138 cells. Secondary virus titer. The median tissue culture infectious dose (TCID50) in (**b**–**d**) was evaluated based on CPE in cells transfected with AS ONs or blank solutions 5 d after infection with SARS-CoV-3 suspension, PIK35 strain (direct virus titer) or media aliquots collected from the infected cells (secondary virus titer). The experiments were performed in 2 biological repeats, 3 replicates each. (**e**) Verification of the antiviral effects of the AS ONs at selected concentrations using RT-qPCR. The virus titer was estimated by the ΔCt method following RNA extraction from the samples in (**c**). * *p*-value < 0.05, two-tailed *t*-test.

None of the ONs showed statistically significant toxicity toward the ACE2-expressing cells (Figure 3a). In ACE2-HEK-293T cells, the control ON had a negligible impact on SARS-CoV-2 replication, while AS ONs G54-LNA and CPS-3 decreased the median tissue culture infectious dose (TCID_50_) by ≥50% at a concentration of 50 nM (*p* < 0.05 for CPS-3) (Figure 3b). The IC_50_ values were equal to 50 ± 30 nM (G54-LNA) and 30 ± 10 nM (CPS-3). In GBM6138 cells (Figure 3c), both AS ONs decreased TCID_50_ by 85 ± 5% at a concentration of 25 nM (*p* < 0.01). The IC50 values were equal to 15 ± 5 nM (G54-LNA) and 4 ± 3 nM (CPE-3). Both AS ONs outperformed the control small molecule antiviral β-d-N4-hydroxycytidine (NHC) [38], which showed moderate activity in GBM6138 cells (IC_50_ = 800 ± 300 nM, Figure 3d) comparable to that observed previously in different cell lines [5,39]. The effects of the AS ONs at concentrations > 12 nM in GBM6138 cells were additionally verified by RT-qPCR assays (Figure 3e). The results agree qualitatively with the CPE assays. Interestingly, in ACE2-HEK-293T, the activity of CPS-3 in the CPE assays was higher than that observed in the DLA (Figure 2e), indicating that additional mechanisms of action (e.g., RNase H activation) could be at play in the case of CPS-3.

We conclude that the leading AS ONs G54-LNA and CPS-3, selected based on EMSA and DLA, inhibited viral replication at subtoxic concentrations within the nanomolar range in HEK-293T-ACE2 and GBM6138. The antiviral activity of CPS-3 must result from a combination of RSF inhibition and RNase H activation or some other mechanisms, while the activity of G54-LNA can be attributed to RSF inhibition exclusively. Future studies including immunoblotting assays are probably needed to obtain conclusive evidence for the dominating mechanism, especially in the case of CPS-3.

## 4. Conclusions

We reported three types of anti-SARS-CoV-2 AS ONs designed to disrupt stem-loops 2/3 of the viral PK and unwind the overall PK structure. Stem 2-targeting AS ONs CPS-3 and G54-LNA (Figure 1b) unwound PK (Figure 2a and Figure S1), inhibited RFS (Figure 2e) in dose-dependent manner and reduced viral replication at nanomolar concentrations in HEK293T-ACE2 and GBM6138 cells (Figure 3b,c). They were superior to analogous AS ONs targeting the supposedly less accessible stem 3 in all in vitro assays (Table 1, Figure 2 and Figure S1). This result supports the importance of the rational target selection for AS ONs.

The stem 2-targeting gapmer G54-LDL unwound PK as efficiently as G54-LNA in EMSA (Figure 2a) but failed to prevent RFS in the luciferase assays (Figure 2c), which was presumably because of the insufficient nuclease resistance. This result points to the importance of the total backbone modification in AS ONs.

The modified AS ON CPS-3 with two G-clamp insertions and the total phosphorothioate backbone modification showed only moderate PK unfolding potential (Figure 2a) and was inferior to G54-LNA in the luciferase assays (Figure 2e) but eventually outperformed G54-LNA in cells exposed to the live virus (Figure 3b,c). This result can be attributed to the fact that unlike LNA, CPS ONs can activate RNase H, which highlights the advantages of dual-effect antivirals.

Thus, although further studies may be required to ascertain the primary molecular mechanisms behind the inhibition of SARS-CoV-2 replication by different PK-targeting AS ONs, the two leading ONs, namely, CPS-3 and G54-LNA, have proved to be potent antivirals in vitro with nanomolar IC_50_ values and could be considered as candidate therapeutics.

## Figures and Tables

**Table 1:** Sequences and activities of AS ONs in EMSA and DLA assays.

Code	Sequence, 5′-3′ ^1^	EC_50_/IC_50_^2^
G54-LDL	**AGC**CCTGTATAC**GAC**	<0.5 µM (EMSA)
A45-LDL	**TAC**GACATCAGT**ACT**	0.6 ± 0.1 µM (EMSA)
G54-LNA	**AGCCCTGTATACGAC**	<0.5 µM (EMSA); 26 ± 9 nM (DLA)
A45-LNA	**TACGACATCAGTACT**	0.3 ± 0.1 µM (EMSA)
CPS-1	*AGCXCTGTATACGAC*	>5 µM (EMSA)
CPS-2	*AGCXCTGTATAXGAC*	>5 µM (EMSA)
CPS-3	*AGXCXTGTATACGAC*	2.3 ± 0.4 µM (EMSA)
CPS-4	*TAXGACATXAGTACT*	>5 µM (EMSA)
CPS-5	*TACGACATXAGTAXT*	>5 µM (EMSA)
SCR	**CATACGTCTATACGCT**	-

^1^ Bold, LNA; Italics, phosphorothioate; X, G-clamp. ^2^ 50% PK unwinding evidenced by electrophoretic mobility shift assay (EMSA) or 50% frameshifting inhibition evidence by dual luciferase assay (DLA).

## Data Availability

All data are provided in the manuscript and the supporting information file.

## Supplementary Materials

The following supporting information can be downloaded at https://www.mdpi.com/article/10.3390/biom13111660/s1, Figure S1: EMSA of PK in the presence of site 1-targeting (blue) or site 2-targeting (red) LNA-DNA-LNA gapmers (LDL) and G-clamp-harboring phosphorothioates (CPS); Figure S2: Verification of PK unwinding by LNA using CD spectrometry; Figure S3: Distribution of AS ONs in HEK293T-ACE2 (a) and GBM6138 (b) cells.
