# Supplementary material for: Unwinding the SARS-CoV-2 Ribosomal Frameshifting Pseudoknot with LNA and G-Clamp-Modified Phosphorothioate Oligonucleotides Inhibits Viral Replication
Source: Biomolecules. 2023 Nov 17;13(11):1660. doi: 10.3390/biom13111660 (PMC10668963; doi:10.3390/biom13111660)
Supplement: Supplementary file 1 [file biomolecules-13-01660-s001.zip › biomolecules-2691468-supplementary.pdf]

Supplementary Materials

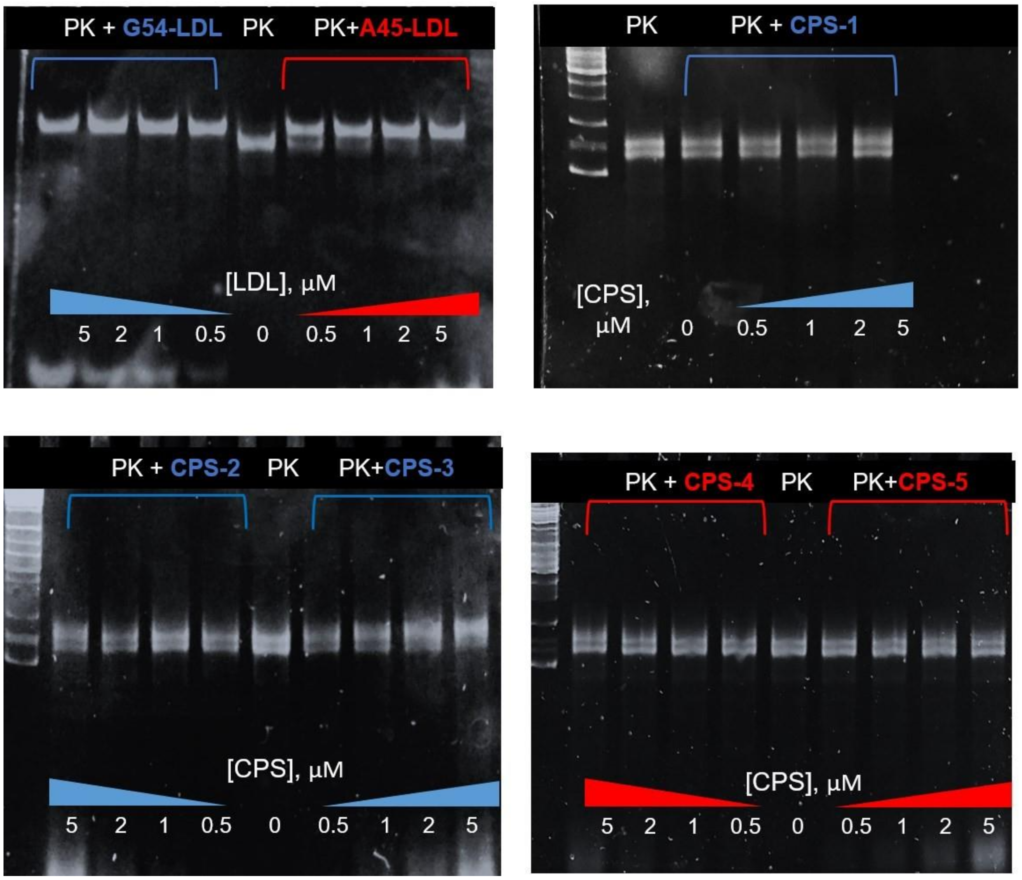

Figure S1. EMSA of PK in the presence of site 1-targeting (blue) or site 2-targeting (red) LNA-DNA-LNA gapmers (LDL) and G-clamp-harboring phosphorothioates (CPS).

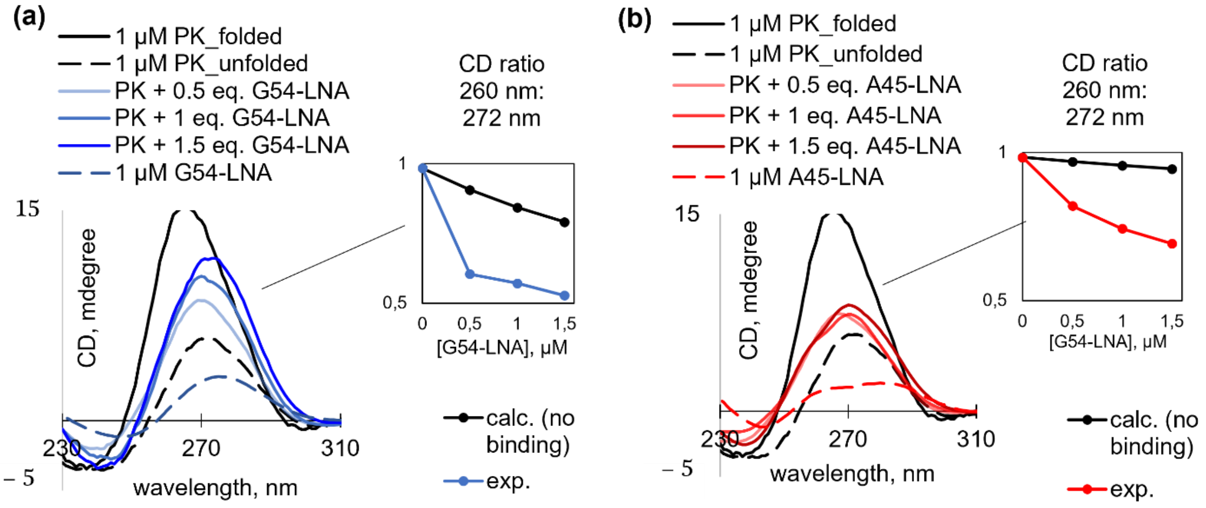

Figure S2. Verification of PK unwinding by LNA using CD spectroscopy. (**a**) Titration of 1uM PK with site 1-targeting G54-LNA (blue). (**b**) Titration of 1uM PK with site 2-targeting A45-LNA (red). CD changes upon LDL-PK interactions and those predicted (calc) for the mixtures with non-binding oligonucleotides.

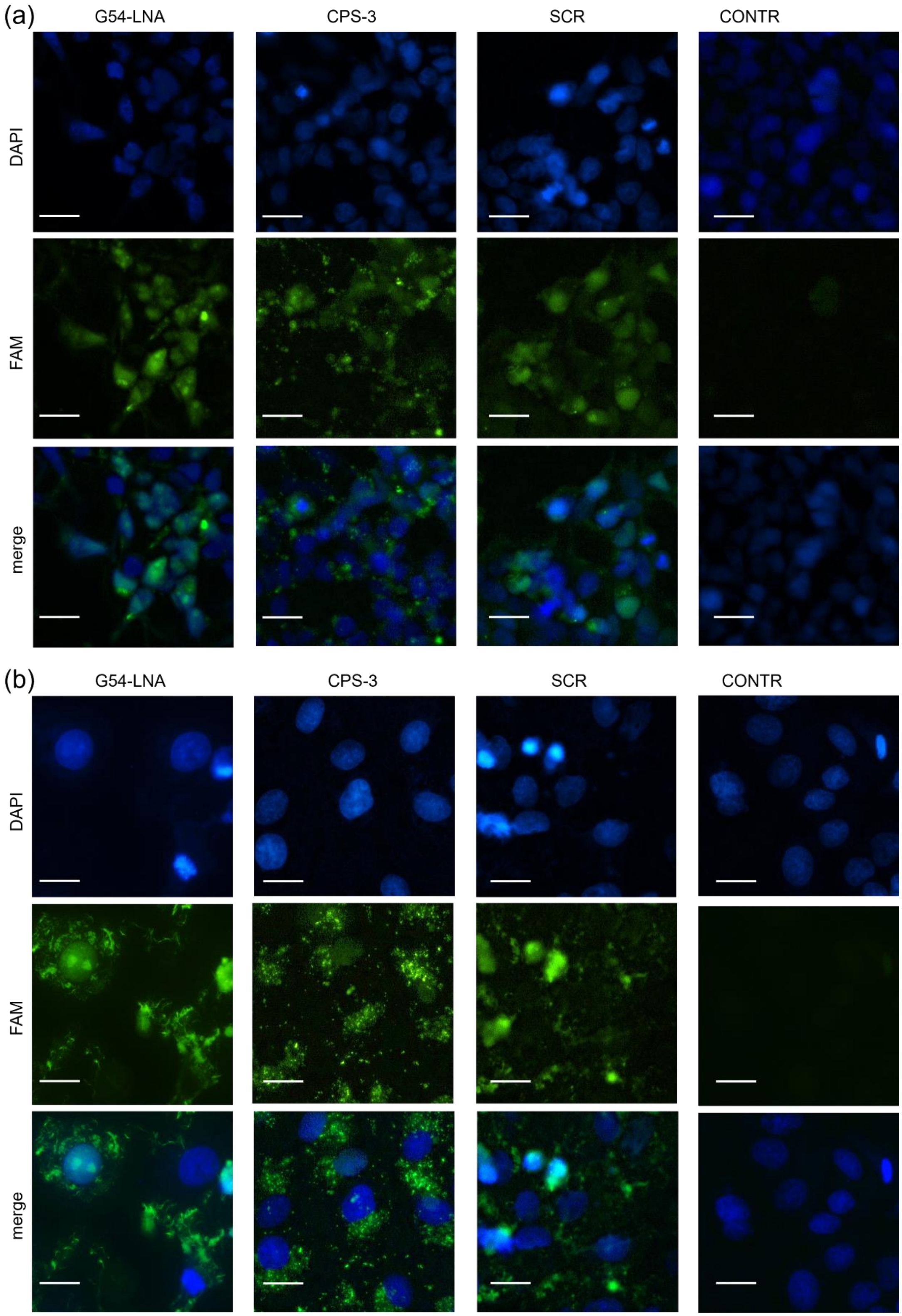

Figure S3. Distribution of AS ONs in HEK293T-ACE2 (**a**) and GBM6138 (**b**) cells. Scale bars: 20 μm.
